# Negligible contribution from aerosols to recent trends in Earth’s energy imbalance

**DOI:** 10.1126/sciadv.adv9429

**Published:** 2025-11-28

**Authors:** Chanyoung Park, Brian J. Soden

**Affiliations:** Rosenstiel School of Marine, Atmospheric, and Earth Science, University of Miami, Miami, FL, USA.

## Abstract

During the 21st century, Earth’s energy imbalance (EEI) at the top of atmosphere has markedly increased because of greater absorbed shortwave (SW) rather than reduced outgoing longwave radiation. Previous studies using single-forcing (aerosol-only) experiments attributed approximately half of the positive SW trend to reductions in anthropogenic aerosols, particularly in the Northern Hemisphere (NH). In contrast, our analysis using observations and reanalysis indicates that both aerosol-radiation and aerosol-cloud interactions have made a negligible contribution to recent EEI trends. While NH anthropogenic aerosols have decreased, enhanced emissions from wildfires and volcanic activity in the Southern Hemisphere (SH) have produced comparable increases, yielding little net global impact. This hemispheric compensation also suggests that model-based estimates may overestimate aerosol influence by overlooking SH aerosol contributions. Despite uncertainties in aerosol proxies, the consistent results from two complementary proxies—satellite-derived aerosol index and reanalysis-based sulfate mass concentration—highlight the importance of accounting for natural source aerosols when assessing EEI trends.

## Teaser

Contrasting aerosol trends between hemispheres cancel out, resulting in minimal impact on Earth’s energy imbalance.

## INTRODUCTION

Earth’s energy imbalance (EEI) at the top of atmosphere (TOA) is a crucial metric for understanding the state of the climate system and an important indicator of climate change (*1*–*4*). It represents the net difference between the amount of solar energy absorbed by Earth and the energy radiated back into space, encompassing both incoming shortwave (SW) and outgoing longwave (LW) radiation. Over the past few decades, observations have revealed an increasing trend in EEI, raising concerns about its potential implications for global climate change [e.g., (*5*, *6*)]. Understanding the factors contributing to this trend is essential for accurately predicting future climate scenarios and for formulating effective mitigation strategies.

Recent studies have highlighted a persistent positive trend in EEI over the past two decades, driven primarily by anthropogenic forcing (*7*–*10*). EEI can be understood as the sum of effective radiative forcing, which includes rapid adjustments to both natural and anthropogenic forcings and the radiative response to these forcings. The latter is influenced by global mean surface temperature changes and the associated climate feedbacks [e.g., (*11*)].

One of the key uncertainties considered in the context of EEI is the role of aerosols. Aerosols, which include both natural and anthropogenic particles suspended in the atmosphere, interact with radiation and clouds through complex physical and chemical processes. These interactions are typically characterized by effective radiative forcing due to aerosol-radiation interactions (ARI) and aerosol-cloud interactions (ACI). ARI, also referred to as aerosol direct effects, involves the influence of aerosols on radiation through scattering and absorption of sunlight [e.g., (*12*)]. ACI, also known as aerosol indirect effects, refers to the modification of cloud properties by aerosols, influencing cloud reflectivity and longevity [e.g., (*13*–*15*)].

Analysis using single-forcing (aerosol-only) experiments indicates that effective radiative forcing due to aerosols exhibits positive trends from 2001 to 2020, driven largely by declines in global aerosol emission in Coupled Model Intercomparison Project phase 6 (CMIP6) historical and SSP2-4.5 scenarios (*9*, *10*). The reduction in anthropogenic aerosol emissions over the Northern Hemisphere (NH) has been identified as a key factor driving the positive trend in EEI, with aerosols’ effective radiative forcing accounting for approximately half of the SWTOA trend (*10*). However, these estimates are model dependent and are not tightly constrained by observations, making it challenging to fully assess aerosols’ impacts on the observed EEI.

To address this gap, our study uses satellite observations and reanalysis data to estimate trends of effective radiative forcing due to aerosols. While previous research has predominantly emphasized the reduction of anthropogenic aerosol emissions in the NH, there has been less attention on the substantial increase in aerosol loading from wildfires and an unexpected volcanic eruption in the Southern Hemisphere (SH) in recent years. These events have introduced large quantities of aerosols into the atmosphere, which provide an interhemispheric contrast with the reductions observed in the NH.

Our findings reveal that the radiative influence of SH aerosols is substantial enough to offset those from aerosol concentration reductions in the NH, leading to a negligible near-global trend in aerosol forcing. This challenges the understanding that declining anthropogenic aerosols in the NH would lead to the positive trend in EEI, suggesting instead that the global impact of aerosols on the EEI trend is minimal, although aerosols have influenced regional variations in the EEI. By providing an observationally based perspective, our research addresses the need for a more accurate understanding of the drivers behind the observed EEI trend. It suggests the importance of factors beyond aerosols, such as natural variability and cloud feedback, in shaping the EEI trend.

## RESULTS

### Observational radiative fluxes

**Fig. 1.**
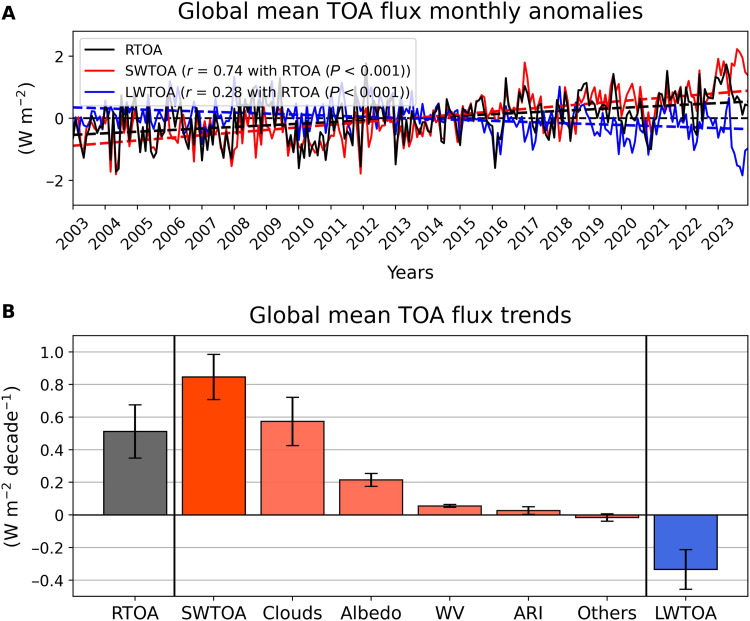
Global mean TOA flux monthly anomalies and trends from 2003 to 2023. (**A**) Global mean monthly anomalies of net RTOA (black solid line), along with the SWTOA (red solid line) and LWTOA (blue solid line) components. Dashed lines show the linear trends for each component. Correlation coefficients (*r*) and associated *P* values between RTOA and SWTOA, as well as RTOA and LWTOA, are provided in the top-left corner. These are calculated from monthly anomalies without removing the long-term trend, thereby reflecting the combined influence of both interannual variability and the persistent trend. (**B**) Global mean TOA flux trends in RTOA and SWTOA, with contributions from changes in clouds, surface albedo, water vapor (WV), ARI, combined effects of trace gases, and solar irradiance (labeled as “others”), as well as trend in LWTOA calculated by observationally based radiative kernel method (*7*, *17*). Error bars represent the 5 to 95% confidence intervals, calculated following the methodology of Santer *et al.* (*18*). Positive anomalies indicate that Earth is absorbing more energy, while negative anomalies represent energy loss.

Figure 1A illustrates the globally averaged monthly anomalies in net TOA radiation (RTOA), SWTOA, and LWTOA, derived from the Clouds and the Earth’s Radiant Energy System (CERES) Energy Balanced and Filled (EBAF) edition (Ed.) 4.2 satellite observational product (*16*). This dataset is known to align well with in situ observational estimates of energy uptake by Earth’s climate system (*6*). The linear trend of RTOA reveals a positive slope of 0.51 ± 0.16 W m^−2^ decade^−1^ (90% confidence interval) from 2003 to 2023, indicating a growing disparity between incoming solar and outgoing terrestrial radiation.

This trend is driven by the radiative imbalance between the SWTOA and LWTOA components. The strong correlation between RTOA and SWTOA (*r* = 0.74, *P* < 0.001) compared to the weaker correlation with LWTOA (*r* = 0.28, *P* < 0.001) reflects the fact that both the interannual variability and the long-term increase in RTOA are dominated by changes in SWTOA. In particular, the pronounced positive trend in SWTOA (0.85 ± 0.14 W m^−2^ decade^−1^ at 90% confidence interval) indicates an enhanced absorption of solar radiation by Earth system. In contrast, the LWTOA exhibits a relatively smaller negative trend of −0.33 ± 0.12 W m^−2^ decade^−1^ (90% confidence interval), which corresponds to an increase in the outgoing LW radiation that partially offsets the SW-driven warming effect but to a lesser extent. The dominance of the SWTOA in this imbalance is leading to a net positive radiative forcing at the top of the atmosphere, contributing to the ongoing global warming trend [e.g., (*6*–*8*, *10*)].

To identify the specific components of SWTOA that contribute to the strong positive trend, we used observation-based radiative kernels from Kramer *et al.* (*7*, *17*). The contributions of the RTOA and LWTOA components are also presented in fig. S1. The SWTOA can be decomposed into contributions from clouds, surface albedo, water vapor, ARI, and “others” (Fig. 1B). The others category includes contributions from solar irradiance and trace gases (*6*). In terms of the global mean, clouds account for 67% (0.57 ± 0.15 W m^−2^ decade^−1^) of the total positive trend in SWTOA (0.85 ± 0.14 W m^−2^ decade^−1^), surface albedo explains 25% (0.21 ± 0.04 W m^−2^ decade^−1^), water vapor contributes 7% (0.06 ± 0.01 W m^−2^ decade^−1^), ARI contribute 3% (0.03 ± 0.02 W m^−2^ decade^−1^), and the remaining −2% (−0.02 ± 0.02 W m^−2^ decade^−1^) is attributed to others. Any changes in SWTOA radiation due to ACI are implicitly included in the cloud contribution. All uncertainties represent 90% confidence intervals and account for autocorrelation in the time series (*18*). These estimates align with the findings of Loeb *et al.* (*6*), who used the observation-based partial radiative perturbation method to decompose SWTOA radiation from September 2002 to March 2020. They also identified strong contributions from clouds and surface albedo to the positive SWTOA trend, while the contribution of aerosol direct effects was negligible (0.01 ± 0.04 W m^−2^ decade^−1^). Therefore, in the remainder of this paper, we will focus more specifically on the portion of the SWTOA cloud contribution attributable to ACI.

### Recent aerosol concentration trends

In this section, we examine the long-term trends in aerosol concentrations using two complementary metrics. The first is the aerosol index (AI) from the moderate resolution imaging spectroradiometer (MODIS) (*19*). AI is calculated as the product of aerosol optical depth (AOD) at 550 nm and the Ångström exponent, which describes the wavelength dependence of AOD and provides insight into aerosol size distribution (e.g., smaller Ångström exponent values indicate larger particles) (*20*). Compared with AOD alone, AI has shown a stronger correlation with cloud condensation nuclei (CCN) (*21*–*23*). The second metric is the sulfate aerosol mass concentration at 925 hPa (SO_4_), derived from the Modern-Era Retrospective Analysis for Research and Applications version 2 (MERRA-2) (*24*, *25*). We include SO_4_ as a proxy for aerosol mass concentration because of its dominant role in cloud droplet formation and ACI, compared to other aerosol types such as black carbon, organic carbon, sea salt, and dust [e.g., (*26*–*28*)].

**Fig. 2.**
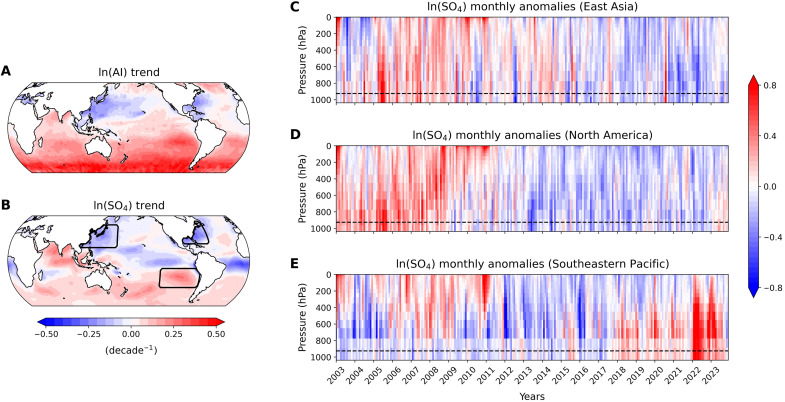
Decadal trends (2003–2023) and monthly anomalies in the natural logarithm of aerosol proxies. (**A**) Spatial maps of trends in the natural logarithm of the AI and (**B**) sulfate aerosol mass concentration at 925 hPa (SO_4_) for the period 2003–2023. (**C** to **E**) Vertical profiles of ln(SO_4_) monthly anomalies over the boxed regions in (B): (C) East Asia, (D) North America, and (E) the Southeastern Pacific. The dashed line in each panel represents the 925-hPa pressure level.

The spatial distribution of trends in the natural logarithm of AI and SO_4_ (Fig. 2, A and B) reveals noticeable regional heterogeneity, with contrasting behaviors observed between the NH and SH. Notably, while both proxies exhibit broad agreement, some differences are expected, as AI captures a wider range of aerosol types beyond sulfate alone. These compositional differences likely account for the observed discrepancies between AI- and SO_4_-based trends (fig. S2).

In the NH, both AI and SO_4_ trends show a marked decrease, particularly over East Asia and North America. By separating AI into its components—AOD at 550 nm and the Ångström exponent—we identify clear signatures of these recent aerosol changes (fig. S3). Near the major industrial regions of East Asia and North America, observations reveal a prominent decline in aerosol abundance (captured by decreasing AOD) accompanied by a consistent shift toward larger particle sizes (indicated by negative Ångström exponent trends). This decline is largely attributed to the implementation of stringent air quality regulations aimed at reducing anthropogenic emissions, especially sulfur dioxide (SO_2_), a key precursor to sulfate aerosols. The reduction in SO_4_ is especially important, given its role in influencing cloud formation and scattering solar radiation. The decreasing SO_4_ trends in these regions are further supported by monthly anomalies in the vertical profiles, which show positive anomalies of SO_4_ until around 2010, followed by a sustained decline in both East Asia and North America (Fig. 2, C and D).

In contrast, the SH presents a different feature, with increasing trends observed in AI and SO_4_, particularly over the Southern Ocean and the Southeastern Pacific, regions typically considered relatively pristine with respect to anthropogenic aerosols. These increases have been less anticipated and have received less scientific attention than NH anthropogenic aerosol reductions. The AI increase in the SH is primarily driven by higher AOD values, accompanied by positive trends in the Ångström exponent—particularly pronounced over the Southern Ocean—indicating a shift toward finer particles (fig. S3). This recent surge in SH aerosol emission is particularly evident in the vertical profiles of sulfate mass concentration anomalies. The Southeastern Pacific (Fig. 2E), in particular, shows noticeable peaks in sulfate concentrations in recent years, which correlates with the timing of multiple wildfires in the SH, including the 2019–2020 Australian wildfires and the 2022 Hunga Tonga–Hunga Ha’apai volcanic eruption (fig. S4). The impact of these events is further amplified by the climatological westerly winds over the Southern Ocean, which facilitate the transport of aerosols emitted in the SH toward the Southeastern Pacific region (*29*). The increase in the frequency and intensity of these aerosol emission events has resulted in sharp, episodic spikes in aerosol concentrations (Fig. 2E). The vertical profiles emphasize the role of wildfires and the unexpected volcanic eruption, coupled with the climatological westerlies, in driving recent aerosol concentration increases in the SH. This also suggests that the SH could play an increasingly important role in global aerosol distribution, particularly as climate change heightens the likelihood of extreme wildfire events (*30*, *31*).

Although SO_4_ is not the primary aerosol emitted by wildfires, atmospheric processes—such as chemical aging, long-range transport, and biogenic precursor production from nutrient deposition—can modify aerosol composition and enhance secondary sulfate formation (*32*–*35*). For example, during the 2019–2020 Australian wildfires, iron-rich pyrogenic aerosols were transported and deposited into the iron-limited Southern Ocean, stimulating unprecedented phytoplankton blooms (*36*). These blooms can increase oceanic emissions of dimethyl sulfide, which is subsequently oxidized in the atmosphere to form additional sulfate aerosols. These biogenically derived sulfates can further contribute to CCN production, as reflected in reanalysis-based CCN estimates (*37*), thereby indirectly amplifying the wildfire influence on regional sulfate loading and potential aerosol-cloud interactions.

### Observational SW ACI estimates

Now, we estimate the SW ACI using an observationally constrained approach, following the method outlined by Park *et al.* (*38*). This approach uses cloud-controlling factor (CCF) analysis for nonobscured low clouds to effectively isolate the influence of aerosols on both cloud droplet number concentrations and their associated radiative effects. The method is also well suited for capturing the impact of highly episodic events, such as the 2019–2020 Australian wildfires and the 2022 Hunga Tonga–Hunga Ha’apai volcanic eruption (see Materials and Methods).

Our satellite observational data have limited coverage over polar regions and face challenges in reliably retrieving ACI over land (*39*–*41*). Therefore, we focus on the oceanic region between 60°S and 60°N as our main domain of analysis to ensure the reliability of our findings. Furthermore, given that most of low-level clouds—those most relevant to aerosol-cloud interactions and, ultimately, to SW ACI (*11*, *42*, *43*)—are concentrated over ocean between 60°S and 60°N, our focus on this domain is well justified (fig. S5).

The SW ACI is estimated using the following equation

$$\begin{array}{c} \text{SW}\;\text{ACI}\;\approx \;\left(\frac{\text{∂SW}\;\_\text{lcld}}{\partial \text{ln}\left(N_{d}\right)}\times \frac{\partial \text{ln}\left(N_{d}\right)}{\partial \text{ln}\left(X\right)}\right)\;\times \;\text{ln}{\left(X\right)}^{\prime } \end{array}$$ (1)

where SW_lcld represents the SW cloud radiative effect from nonobscured (nonoverlapped) low-level clouds, obtained from the CERES FluxByCldTyp Ed. 4.1 dataset (*44*). *N*_d_ represents cloud droplet number concentration, derived from MODIS satellite observations. *X* denotes the aerosol concentration proxy, taken as either AI or SO_4_. Primes indicate monthly anomalies relative to the climatological seasonal cycle.

The right-hand side of the equation consists of two main components. The first component is the susceptibility of the SW low-cloud radiative effect to variations in aerosol concentration, derived from CCF analysis while holding other environmental influences constant (*38*, *45*, *46*). We apply the cloud radiative kernel to quantify the radiative response solely to changes in cloud properties, excluding contributions from noncloud factors. In this framework, the susceptibility explicitly incorporates the aerosol activation rate $\left[\partial \text{ln}\left(N_{d}\right)\;/\;\partial \text{ln}\left(X\right)\right]$ , thereby linking aerosol changes to cloud droplet number concentration and, in turn, to SW cloud radiative effects. The second component is the corresponding monthly anomalies in aerosol concentration relative to the climatology for the given time period. Further details regarding this approach and the equation can be found in the work of Park *et al.* (*38*).

**Fig. 3.**
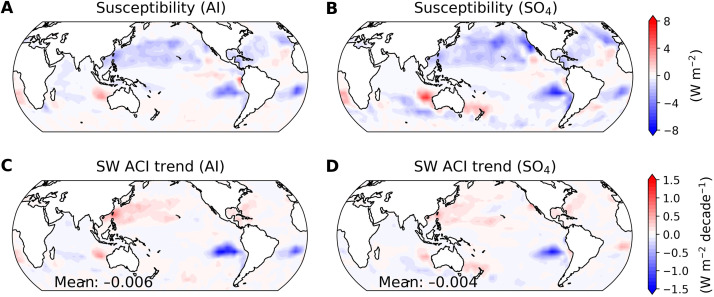
Spatial distributions of nonobscured low-cloud susceptibility to variations in aerosol concentrations and decadal trends (2003 to 2023) in SW effective radiative forcing from ACI, differentiated by aerosol proxies. (**A**) Susceptibility for AI. (**B**) Same as (A), but for SO_4_. (**C**) Observationally constrained SW ACI trend for AI from 2003 to 2023. (**D**) Same as (C), but for SO_4_. The domain-averaged (60°S to 60°N, ocean) SW ACI trends are shown in the bottom-left corners of each panel.

Figure 3 (A and B) shows the susceptibility for both AI and SO_4_, indicating that increases in aerosol concentrations are associated with negative anomalies of SW_lcld across most of the global ocean, particularly in regions dominated by low clouds, such as the mid-latitudes in the NH and the Southeastern Pacific. The strong negative susceptibility in these regions reflects the Twomey effect, where more aerosols produce smaller, more numerous droplets that increase cloud albedo and enhance SW reflection, thereby exerting a cooling effect (*13*). In addition, smaller droplets suppress precipitation and extend cloud lifetime, further sustaining the cooling influence (*14*, *15*). In contrast, some regions with positive susceptibility indicate that higher aerosol concentrations reduce cloud droplet size in a way that enhances their evaporation during dry-air entrainment. Smaller droplets also sediment more slowly, increasing exposure to entrained air and accelerating liquid water loss, which thins clouds and reduces albedo (*47*, *48*).

By multiplying the susceptibility with monthly anomalies of aerosol proxies, the resulting SW ACI trends further illustrate the regional patterns in aerosol-cloud interactions (Fig. 3, C and D). We observe positive SW ACI trends near East Asia and North America, where anthropogenic aerosol emissions have declined, while strong negative SW ACI is evident in the Southeastern Pacific, driven primarily by increased aerosol concentrations due to wildfires and the volcanic eruption in the SH. The domain-averaged (60°S and 60°N, over ocean) values for both proxies indicate slight negative trends, with a mean value of −0.006 ± 0.028 W m^−2^ decade^−1^ for AI and −0.004 ± 0.025 W m^−2^ decade^−1^ for SO_4_ at the 90% confidence interval. This value is sufficiently small to be considered negligible when compared with the global SW cloud component (0.57 ± 0.15 W m^−2^ decade^−1^ at 90% confidence interval).

These results highlight the interplay between regional susceptibility and aerosol concentration changes in shaping the SW ACI trends. In particular, the strong negative susceptibility in the southeastern Pacific, combined with increasing aerosol loading from wildfires and volcanic activity, produces a radiative cooling effect in the SH. This SH cooling is of comparable magnitude to the radiative warming associated with aerosol reductions in the NH, highlighting the hemispheric asymmetry in ACI and the compensating role of increased SH aerosol concentrations in modulating the global radiative balance.

### Comparison with CMIP6 SW ACI

We next investigate the SW ACI trends over the 2003 to 2023 period using outputs from five models participating in the Radiative Forcing Model Intercomparison Project (RFMIP) (*49*) single-forcing (aerosol-only) experiments. These experiments capture genuine aerosol-cloud interactions that are unaffected by changes in sea surface temperature (SST). To estimate the models’ SW ACI, we use the following simplified equation, which was also used to validate the observationally constrained ACI, as presented by Park *et al.* (*38*)

$$\text{SW}\;\text{ACI}=\text{SW}\;\_{\text{lcld}}^{\prime }$$ (2)

where the low-level SW cloud radiative response ( $\text{SW}\;\_{\text{lcld}}^{\prime }$ ) is determined using the cloud classification method introduced by Webb *et al.* (*50*) and Soden and Vecchi (*51*). The radiative kernel technique is applied to isolate the cloud response to aerosols, thereby excluding contributions from noncloud components (see Materials and Methods).

**Fig. 4.**
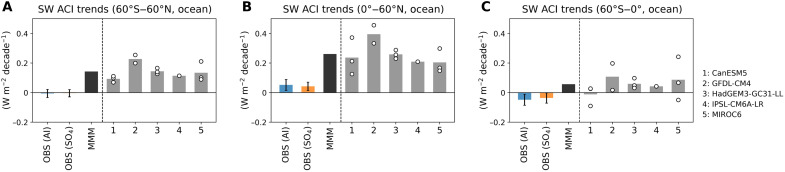
Decadal trends (2003–2023) in SW ACI over oceans across three different domains. (**A**) Near-global (60°S to 60°N), (**B**) NH (0° to 60°N), and (**C**) SH (60°S to 0°). Observationally (OBS) constrained SW ACI estimates are derived from two aerosol proxies: AI (blue) and SO_4_ (orange). Uncertainties are calculated by combining the methods from Park *et al.* (*38*) and Santer *et al.* (*18*) (see Materials and Methods). The MMM (black) is derived from five models in the RFMIP (*49*) single-forcing (aerosol-only) experiments. Individual model realizations are depicted as hollow circles, with gray bars representing the mean of these realizations.

Figure 4 presents the decadal SW ACI trends especially over oceans for three key domains: near-global (60°S to 60°N), NH (0° to 60°N), and SH (60°S to 0°). On a near-global scale (Fig. 4A), the observational estimates for both AI and SO_4_ suggest near-zero trends (−0.006 ± 0.028 W m^−2^ decade^−1^ for AI and −0.004 ± 0.025 W m^−2^ decade^−1^ for SO_4_ at 90% confidence interval), indicating minimal change in ACI over the past two decades. In contrast, the multimodel mean (MMM) from five models shows a much stronger positive trend (0.14 W m^−2^ decade^−1^). When examining each model and its realizations individually, the positive values are notably stronger compared to the observational estimates. To better understand the global trends, it is crucial to assess the contributions from each hemisphere separately.

In the NH (Fig. 4B), the RFMIP models project a strong positive SW ACI trend (0.26 W m^−2^ decade^−1^ for MMM), driven primarily by areas near industrial regions such as East Asia and North America, where anthropogenic aerosol emission reductions have been observed (fig. S6). However, the observational estimates reveal a smaller positive trend (0.052 ± 0.037 W m^−2^ decade^−1^ for AI and 0.04 ± 0.029 W m^−2^ decade^−1^ for SO_4_ at 90% confidence interval). This discrepancy suggests that the SSP2-4.5 aerosol emission scenario, which serves as the base scenario for RFMIP experiments post-2014, may overestimate the reduction of aerosol concentrations in the NH compared to its actual values.

This result is further supported by fig. S7, which illustrates the monthly anomalies of aerosol concentrations from observations and reanalysis compared to those projected under the historical plus SSP2-4.5 scenario. In the NH, the observed aerosol reduction slopes are −0.014 for AI and −0.041 for SO_4_, while the MMM shows steeper declines of −0.144 for AI and −0.217 for SO_4_, overestimating aerosol concentration reductions by at least a factor of 5 (fig. S7, C and D).

The SH presents more complex features (Fig. 4C). Observational estimates indicate negative SH SW ACI trend (−0.048 ± 0.037 W m^−2^ decade^−1^ for AI and −0.037 ± 0.035 W m^−2^ decade^−1^ SO_4_ at 90% confidence interval), which is attributed to the observed increase in aerosol concentrations from wildfires and volcanic activity, particularly in the Southeastern Pacific, where SW cloud radiative effects exhibit strong negative susceptibility to aerosols. In contrast, the MMM from the models suggests a positive trend (0.06 W m^−2^ decade^−1^), with a broad positive spatial distribution across the SH (fig. S6). These differences between observation-based and model estimates reflect the absence of aerosol emissions from wildfires or volcanic activity in the models, which are not incorporated into future projections. Some individual model realizations even exhibit inconsistent signs of SH SW ACI trends (Fig. 4C).

The contrasting aerosol concentration trends between observations and model outputs provide further support for these findings (fig. S7, E and F). Noticeable increases in aerosol concentrations are observed following wildfire and the volcanic eruption events, while models predict near-neutral aerosol concentration trends. These discrepancies emphasize the need to account for aerosols emitted from wildfires and volcanic eruptions, as well as their interactions with clouds when interpreting recent EEI changes. In particular, the observed increase in SH aerosols, contrasted with the reductions in the NH, points to an emerging hemispheric asymmetry in global aerosol-cloud interactions.

**Fig. 5.**
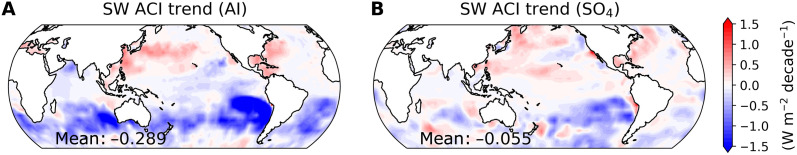
Spatial distributions of observationally constrained SW ACI trends from 2003 to 2023, using an alternative method, as described by Wall *et al.* (*46*). (**A**) SW ACI trend for AI. (**B**) Same as (A), but for SO_4_. The domain-averaged (60°S to 60°N, ocean) SW ACI trends are shown in the bottom-left corners of each panel.

To assess the robustness of our observational estimates, we apply an alternative observationally constrained SW ACI estimation method introduced by Wall *et al.* (*46*), which has also been validated for its applicability to episodic events. This approach is similar to Eq. 1 but does not incorporate the activation rate. The results provide additional evidence of the impact of increased aerosol concentrations in the SH, with a near-global average of −0.289 ± 0.12 W m^−2^ decade^−1^ for AI and −0.055 ± 0.075 W m^−2^ decade^−1^ for SO_4_ at 90% confidence interval (Fig. 5). These findings reveal an increase in SW ACI trend in the NH (0.057 ± 0.11 W m^−2^ decade^−1^ for AI and 0.085 ± 0.074 W m^−2^ decade^−1^ for SO_4_ at 90% confidence interval), contrasted by more pronounced negative SH SW ACI trends of −0.52 ± 0.2 W m^−2^ decade^−1^ for AI and −0.15 ± 0.1 W m^−2^ decade^−1^ for SO_4_ (both at the 90% confidence level). Together, these results indicate an overall negative contribution of ACI to recent EEI trends. The persistence of this hemispheric compensation even under different baseline assumptions and susceptibility in SW ACI estimation reinforces the robustness of our conclusion. These results demonstrate that the observed compensation is not an artifact of methodological choice but rather a consistent feature across approaches.

We note, however, that Wall *et al.* (*46*) exclusively used SO_4_ as the aerosol proxy, whereas applying their method with AI produces a stronger negative SW ACI trend. This likely reflects an overestimation, particularly in the SH, since AI is a column- and species-integrated metric that combines all aerosol types—including episodic wildfire and volcanic emissions—rather than isolating sulfate. This integration introduces uncertainty when applied in a framework designed to evaluate sulfate-specific forcing. Moreover, AI exhibits a weaker activation rate to *N*_d_ than SO_4_ in regions such as the Southern Ocean (*38*), raising the potential for regional biases if activation rate is not explicitly accounted for. By contrast, our use of sulfate mass concentration at 925 hPa better represents the dominant aerosol type and conditions relevant to cloud formation near cloud base (*26*–*28*, *52*), but it excludes other aerosol types such as organic and black carbon, whose emissions are increasing because of intensified wildfire activity (fig. S2). These omissions likewise add uncertainty to the magnitude of SW ACI trends. Nevertheless, both AI- and SO_4_-based approaches consistently reveal hemispheric compensation, with declining anthropogenic aerosols in the NH offset by natural aerosol concentration increases in the SH. The complementary strengths of these proxies reinforce the robustness of our conclusion that ACI have made a negligible net contribution to the recent EEI trends.

## DISCUSSION

We examined how aerosols contribute to the recent trends in EEI through two mechanisms: ARI and ACI. Despite the recognized warming effects from reduced aerosol concentrations in the NH, the concurrent increase in aerosol concentrations in the SH appears sufficient to counterbalance the warming effects observed in the NH. As a result, while aerosols have played a role in regional variations in EEI, their overall contribution to global EEI trends has been minimal over the past few decades.

MODIS satellite orbit drift, beginning in early 2020 for Terra and early 2021 for Aqua (*53*), may have influenced our estimates. In addition, two extreme aerosol emission events in the SH—the 2019–2020 Australian wildfires and the 2022 Hunga Tonga–Hunga Ha’apai eruption—could influence decadal trend estimates. To assess the sensitivity of our results to these potential biases, we repeated the analysis for a shorter period (2003 to 2018), thereby excluding the years impacted by orbit drift and these extreme episodic events. Although this truncated period omits key features of the recent record, both aerosol concentration trends and SW ACI results remain consistent (fig. S8). Specifically, we still observe pronounced aerosol concentration reductions in the NH, increases in the SH, and a negligible SW ACI trend over the domain (60°S and 60°N, over ocean) of −0.01 ± 0.028 W m^−2^ decade^−1^ for AI and 0.022 ± 0.027 W m^−2^ decade^−1^ for SO_4_ (90% confidence interval). This consistency supports the robustness of our findings against potential biases from orbit drift and extreme events.

To further support our conclusions, we also estimate ACI using SO_4_ from the Copernicus Atmosphere Monitoring Service (CAMS) reanalysis (*54*) (fig. S9). This additional analysis allows us to evaluate whether our findings hold when using a different observationally constrained reanalysis dataset that incorporates comprehensive assimilation of satellite and ground-based measurements. Despite stronger ACI in the NH, the near-global contribution to EEI remains negligible (0.058 ± 0.031 W m^−2^ decade^−1^ at the 90% confidence level). The enhanced NH signal may be influenced by the use of RCP8.5-based emission projections in the reanalysis beginning around 2010, which prescribe a substantial and rapid reduction in sulfur dioxide emissions and, consequently, sulfate mass concentrations (fig. S10B) (*54*, *55*).

We also incorporate results from the RFMIP piClim-histnat experiments, which are specifically designed to account for natural sources of aerosol emissions, such as volcanic eruptions, with a particular emphasis on stratospheric aerosols. When examining ACI in these experiments, models estimate a slightly negative trend in NH SW ACI of −0.059 W m^−2^ decade^−1^. In contrast, the contribution in the SH is negligible, with a trend of −0.004 W m^−2^ decade^−1^ (fig. S11). Overall, single-forcing experiments (both aerosol-only and natural forcing–only), which use fixed SST, fail to adequately represent the observed changes in aerosol concentrations and the influences of ACI on EEI, despite the limited number of models involved in these experiments.

While our focus has been on aerosols, it is important to acknowledge that other components, such as natural variability and cloud feedback may play dominant roles in shaping the global EEI. For instance, the transition to a positive phase of the Pacific Decadal Oscillation (PDO) in 2014 has been linked to changes in SST and cloud cover, which likely contributed to variations in EEI (*6*, *56*, *57*). This phase was associated with pronounced SST warming in the eastern Pacific that persisted through 2020, accompanied by reductions in low-cloud cover that enhanced SW absorption and amplified the warming (*56*). Although the PDO index returned to negative values around 2017, SSTs have remained anomalously high through 2023, indicating that PDO-related variability alone cannot explain the sustained EEI trend. Instead, its influence appears transient, superimposed on longer-term warming driven by other processes.

One such process is the reduction in SW reflection from positive SW cloud feedback, identified by Raghuraman *et al.* (*9*) as one of the primary drivers of the recent EEI increase. This contrasts with LW radiation, where negative cloud feedback acts to partially stabilize the system by offsetting some of the imposed radiative perturbations. In the SW, however, no comparable compensating feedback exists, and, thus, reductions in cloud reflectivity directly amplify the EEI, leading to continuous heat accumulation and contributing to the persistent increasing trend in EEI. Nevertheless, substantial uncertainties remain in quantifying the magnitude of SW cloud feedback. Observational estimates vary widely depending on the datasets, methods, and time periods analyzed, with some studies suggesting values near zero, while others indicating pronounced positive feedback (*9*, *58*–*61*). These uncertainties reflect persistent challenges in constraining cloud processes and their response to SST patterns, limiting our ability to tightly constrain the exact contribution of SW cloud feedback to the EEI trend. Even so, the absence of compensating mechanisms in the SW underscores its potential to play a disproportionate role in sustaining the long-term increase in EEI.

## MATERIALS AND METHODS

In this study, we restrict our analysis to monthly temporal resolution from January 2003 to December 2023, focusing on the geographical coverage spanning from 60°S to 60°N over the ocean, due to unreliable retrievals of satellite observations over land and polar regions (*39*–*41*). All data fields were interpolated onto a 2.5° by 2.5° grid.

### Observation and reanalysis data

We use various datasets from the CERES for our analysis. To calculate the global trend in TOA radiative fluxes, we use the CERES EBAF Ed. 4.2 satellite observational product (*16*). For the estimation of SW ACI, we rely on the CERES FluxByCldTyp Ed. 4.1 dataset (*44*), which allows us to focus on nonobscured (nonoverlapped) low-level clouds, where ACI are most relevant (*11*, *42*, *43*). For SW ARI, we use the CERES EBAF Ed. 4.2 dataset in combination with the CERES SYN1deg Ed. 4A product (*62*). However, the latter is only used to estimate aerosol direct effects under clear-sky conditions, as cloud properties derived from geostationary satellites in the SYN1deg product contain artifacts that limit their accuracy in cloudy-sky conditions.

We use the MODIS (*19*) data from both the Aqua and Terra satellites (MOD08_M and MYD08_M, respectively) for the AI, which serves as a proxy for aerosol concentration. By combining datasets from the two satellites, we enhance the robustness of our analysis. The AI is derived from the product of AOD at 550 nm and the Ångström exponent, the latter of which reflects the wavelength dependence of AOD, providing insights into aerosol size distribution (e.g., a smaller Ångström exponent indicates larger particles) (*20*). AI has shown a stronger correlation with CCNs compared to AOD alone (*21*–*23*). Nevertheless, AI observations remain affected by near-cloud retrieval artifacts (*63*). We also note that the positive AOD trend over the Southern Ocean observed in MODIS (fig. S3) is not reproduced by the Multiangle Imaging SpectroRadiometer (MISR) (*64*). However, MISR’s limited sampling frequency, providing fewer than 2 days of coverage per month in many regions (*65*), raises concerns about its ability to represent monthly means. In contrast, MODIS offers much more continuous coverage, with at least 15 days of observations per month globally (*65*), making it better suited for robust analyses of monthly-to-decadal trends.

We also use the MERRA-2 reanalysis (*24*, *25*). MERRA-2 integrates observations with global model simulations to provide estimates of atmospheric conditions. For example, the total AOD is observationally constrained using MODIS satellite data, and the distribution and vertical profiles of different aerosol species are model derived. Since AI from MODIS provides aerosol species-integrated, column-integrated quantities and does not account for the vertical profile, it may not accurately capture aerosol-cloud interactions, which mainly occur in low-level clouds. To better represent aerosols relevant for these interactions, we use sulfate mass concentration at 925 hPa (SO_4_) instead of surface level as our reference, as conditions near 925 hPa better reflect CCN concentrations at the cloud base (*52*). These data are obtained from the 3-hourly MERRA-2 file “inst3_3d_aer_Nv” and averaged to monthly resolution for analysis.

In addition, we use the CAMS reanalysis (*54*), which assimilates satellite retrievals—most notably, MODIS AOD—but, similar to MERRA-2 reanalysis, relies on its own atmospheric modeling to partition aerosol species and represent their vertical distribution. CAMS reanalysis integrates these observations into the Integrated Forecasting System, providing global, time-consistent fields for sulfate, black carbon, organic carbon, dust, and sea salt. Although the total AOD from MODIS is observationally constrained, the relative contributions of each aerosol type and their vertical profiles are model derived, meaning that the representation of low-level aerosols relevant for cloud interactions remains dependent on the reanalysis system. For consistency with MERRA-2, we use sulfate mass concentration at 925 hPa as the proxy for cloud-relevant aerosols. Including CAMS reanalysis alongside MERRA-2 enables us to test the robustness of our findings to uncertainties stemming from the choice of reanalysis product and assimilation framework.

We use cloud droplet number concentration (*N*_d_) provided by Gryspeerdt *et al.* (*40*), which was calculated from MODIS cloud optical depth and effective radius. Data from both the Terra and Aqua satellites are combined. *N*_d_ measurements can be subject to biases under specific conditions (*66*–*68*). To enhance the accuracy and reliability of our *N*_d_ retrievals, we apply a rigorous sampling strategy. This approach selects only single-layer liquid clouds that meet predefined criteria, known as the “BR17 sampling” method, as presented by Gryspeerdt *et al.* (*40*). These criteria, introduced by Bennartz and Rausch (*69*), demonstrate the highest correlation with aircraft data. However, the use of different sampling methods introduced by Gryspeerdt *et al.* (*40*) does not affect our conclusions. In addition, this dataset was not filtered to only include low-level clouds, which may have weakened the connection between surface aerosol sources and *N*_d_ (*28*).

### CMIP6 data

Previous studies have used CMIP6 models to estimate aerosol contributions to the recent EEI trend, making it essential to compare our observational estimates of ACI with those derived from CMIP6 models. To assess the true aerosol effect on the recent EEI, we use single-forcing (aerosol-only) experiments from the RFMIP (*49*), specifically the piClim-histaer experiments. These experiments prescribe SSTs and sea ice conditions based on a preindustrial climatology while following historical aerosol emissions through 2014 and the SSP2-4.5 scenario thereafter. We examine five models that provide extended simulations through 2023, including their available realizations.

Although the piClim-histnat experiment accounts for the influence of volcanic eruptions, it primarily focuses on stratospheric aerosol concentrations from volcanic activity. Therefore, in this study, we use the piClim-histaer experiment as our primary reference for estimating SW ACI. To evaluate the influence of volcanic aerosols, we additionally analyze the first realization (r1) of piClim-histnat experiments from the same five models.

### Radiative kernel techniques

Radiative kernels, first introduced by Soden and Held (*70*) to analyze radiative feedbacks, quantify the differential response of radiative fluxes to small perturbations in key state variables such as clouds, surface albedo, temperature, and water vapor. This allows us to isolate the true cloud radiative response without interference from cloud masking effects. In this study, radiative kernels are applied to deseasonalized monthly anomalies from 2003 to 2023, calculated as deviations from the mean of that period.

For our observational analysis, we use radiative kernels derived from CloudSat and the Cloud-Aerosol Lidar and Infrared Pathfinder Satellite Observation (CALIPSO) data (*7*, *17*). Radiative flux anomalies are sourced from the CERES EBAF Ed. 4.2 product (*16*). Temperature and specific humidity anomalies are obtained from the ERA5 reanalysis (*71*), while surface temperature data are from the GISS Surface Temperature Analysis version 4 (GISTEMP v4) (*72*). Contributions from others factors—including solar irradiance and trace gases—are estimated as residuals from all other components (e.g., clouds, surface albedo, water vapor, and aerosol direct effects) in the total SWTOA. Since aerosol direct effects are not included in our radiative kernel, we estimate them separately as detailed in the “Estimating SW ARI” section.

For our model analysis, we use radiative kernels derived from the HadGEM3-GA7.1 model (*73*) for CMIP6 model simulations. The HadGEM3-GA7.1 kernel is representative of the commonly used radiative kernels in the literature for tropospheric and surface adjustments (*73*). Differences introduced by using different kernels are less than 0.1 W m^−2^ (*74*, *75*).

### Estimating SW ARI

To estimate the aerosol direct effects, it is crucial to consider the influence of cloud presence, including factors such as cloud height relative to aerosol layers. These factors influence the radiative effects of aerosols. Aerosols located above clouds reduce cloud reflectivity, leading to a relative warming at the TOA, which has a much larger impact compared to the surface. Conversely, when clouds are positioned above aerosols, they can block aerosol interactions with solar radiation (*76*–*78*).

In this study, we classify sky conditions as either cloudy or clear to capture these variations in radiative effects at the TOA. The contribution of each aerosol direct effect is weighted by both the cloud fraction and the clear-sky fraction (*79*). Our analysis primarily uses CERES product, and the estimation of SW ARI is based on the following equation

$$\text{SW}\;\text{ARI}={\left(\text{SW}\;\text{DR}E_{\text{cld}}\times \text{CF}\right)}^{\prime }+{\left(\text{SW}\;{\text{DRE}}_{\text{clr}}\times \left(1-\text{CF}\right)\right)}^{\prime }$$ (3)

where SW DRE_cld_ refers to the SW aerosol direct radiative effects (DREs) under cloudy-sky conditions, SW DRE_clr_ refers to the SW DRE under clear-sky conditions, and CF represents the cloud fraction. The first term on the right-hand side represents the monthly anomalies of the aerosol direct effect under cloudy sky, weighted by cloud fraction, while the second term represents the effect under clear sky, weighted by clear-sky fraction. Primes denote monthly anomalies relative to the climatological seasonal cycle.

Estimating aerosol direct effects requires a radiative transfer model to assess the difference between conditions with and without aerosols. The estimate of SW DRE_cld_ relies on MERRA-2, whereas SW DRE_clr_ is estimated using the method described in Loeb *et al.* (*80*), which combines calculated fluxes from the CERES SYN1deg Ed. 4A dataset (*62*) with observed fluxes from the CERES EBAF Ed. 4.2 (*16*). This approach accounts for the masking effect of aerosols on surface albedo variations caused by the presence of aerosols.

Using MERRA-2 reanalysis data for SW DRE_cld_ while relying on CERES for other components may introduce some uncertainty into our SW ARI estimates, as cloudy-sky conditions can modulate the radiative forcing of aerosols differently compared to clear-sky conditions. However, given that ARI have been shown to exert a relatively negligible influence on the trend in EEI in both this study and previous study (*6*), the overall uncertainty introduced by this limitation is not expected to alter our conclusions.

### Estimating SW ACI and validating with episodic events

To estimate SW ACI independently of environmental factors that influence cloud droplet number concentration, low-cloud properties, and their associated radiative effects, we apply a CCF analysis following the framework established by Scott *et al.* (*45*) and Park *et al.* (*38*). This approach incorporates a set of environmental variables known to modulate cloud microphysical properties and low-cloud behavior. The relationships are expressed as follows

$$\text{SW}\;\_{\text{lcld}}^{\prime }\approx \sum_{i=1}^{7}\frac{\text{∂SW}\;\_\text{lcld}}{\partial Y_{i}}\times {Y_{i}}^{\prime }$$ (4)

$$\begin{array}{c} \text{ln}{\left(N_{d}\right)}^{\prime }\approx \sum_{i=1}^{6}\frac{\partial \text{ln}\left(N_{d}\right)}{\partial Y_{i}}\times {Y_{i}}^{\prime }+\frac{\partial \text{ln}\left(N_{d}\right)}{\partial \text{ln}\left(X\right)}\times \text{ln}{\left(X\right)}^{\prime } \end{array}$$ (5)

where SW_lcld represents the nonobscured low-level SW cloud radiative effect from CERES FluxByCldTyp Ed. 4.1 dataset (*44*). The controlling factors ( $Y_{i}$ ), taken from MERRA-2 reanalysis, include (i) SST, (ii) estimated inversion strength, (iii) horizontal surface temperature advection, (iv) relative humidity at 700 hPa, (v) vertical velocity at 700 hPa, and (vi) near-surface wind speed. These variables represent a combination of thermodynamic and dynamic processes that are critical for low-cloud formation and persistence (*45*). In addition, we include (vii) MODIS-derived cloud droplet number concentration (*N*_d_), expressed on a natural logarithmic scale, as a controlling factor to explicitly account for the aerosol activation rate (*38*). For the aerosol concentration proxy *X*, we use either the AI from MODIS or SO_4_ from the MERRA-2 reanalysis. To ensure that the marine boundary layer has sufficient time to adjust to the ambient meteorological environment, we average the dataset to a 5° by 5° grid using cosine-of-latitude area weighting (*45*, *81*, *82*).

At each grid point, we apply ordinary least-squares multiple linear regression to model SW_lcld′ or ln(*N*_d_)′ against anomalies in the CCFs, with both predictors and response variables detrended and deseasonalized. The regression model is trained on data from January 2003 to December 2018, with periods affected by MODIS satellite orbit drift—beginning in early 2020 for Terra and early 2021 for Aqua (*53*)—and two major aerosol emission events—the 2019–2020 Australian wildfires and the 2022 Hunga Tonga–Hunga Ha’apai volcanic eruption—fully excluded to prevent potential bias. The trained regression model is then interpolated to a 2.5° by 2.5° grid. In this study, we focus specifically on the contribution of aerosol concentration variations to ln(*N*_d_)′ and their subsequent influence on SW_lcld′, expressed as susceptibility [∂ SW_lcld/∂ ln(*N*_d_)] × [∂ ln(*N*_d_)/∂ ln(*X*)], while holding all other environmental conditions constant.

The opportunistic experiment evaluates whether our method can predict anomalies in SW_lcld during the two extreme episodic aerosol emission events. The analysis focuses on the southeastern Pacific, a region that experienced increased aerosol concentrations associated with both events (Fig. 2). For each event—October 2019–May 2020 (wildfires) and January–November 2022 (volcanic eruption)—predictor variables are temporally averaged over the respective event periods and input into the trained regression model to predict the corresponding SW_lcld anomalies. Both predicted and observed anomalies are then spatially averaged over the southeastern Pacific for comparison. Our method successfully captures the regional mean of SW_lcld′ during both events, demonstrating its skill in predicting low-cloud radiative anomalies driven by changes in aerosol concentrations (fig. S12). This supports the suitability of our methodology for estimating SW ACI, even under episodic high-aerosol emission conditions.

### Uncertainty from estimating SW ACI trend

Unlike aerosol concentration trends observed directly, estimating ACI introduces additional uncertainties due to the complex calculations involved in the estimation process. Therefore, we account for these uncertainties by combining those arising from susceptibility with those from the observed aerosol concentration trend.

To quantify the uncertainty in the regression coefficients of susceptibility, a 90% confidence interval for susceptibility at each grid box is calculated as follows

$$\varepsilon =t_{\mathrm{crit}}\sqrt{∆x^{T}C_{\text{ii}}∆x}\sqrt{\frac{N_{\text{nom}}}{N_{\text{eff}}}}\frac{d\text{ln}\left(X\right)}{\mathrm{dt}}$$ (6)

where $t_{\mathrm{crit}}$ is the critical value of the Student’s *t* test at the 95% confidence level with $N_{\text{eff}}-7$ degrees of freedom (*83*). Δx is the regression coefficient for $\partial \text{ln}\left(N_{d}\right)/\partial \text{ln}\left(X\right)$ , where *X* represents either AI or SO_4_. C represents the variance-covariance matrix of regression coefficients, with diagonal components $C_{\text{ii}}$ , given by $C={\hat{\sigma }}^{2}{\left(Z^{T}Z\right)}^{-1}$ , where ${\hat{\sigma }}^{2}$ denotes the mean of squared residuals of the regression model and Z is the data matrix composed of detrended monthly anomalies of $\text{ln}\left(N_{d}\right)$ . The ratio $N_{\text{nom}}/N_{\text{eff}}$ corrects for autocorrelation and is estimated as (1+r)/(1−r) , where r is the lag one autocorrelation of monthly anomalies of SW_lcld . Last, dln(X)/dt denotes the trend of aerosol proxy over 2003 to 2023.

Uncertainty for spatially averaged regression coefficients is calculated as

$${∆}_{\text{susceptibility}}=\sqrt{\frac{\sum_{k=1}^{N_{\text{nom}}^{*}}{\left(\varepsilon_{k}w_{k}\right)}^{2}}{{\left(\sum_{k=1}^{N_{\text{nom}}^{*}}w_{k}\right)}^{2}}}\sqrt{\frac{N_{\text{nom}}^{*}}{N_{\text{eff}}^{*}}}$$ (7)

where $\varepsilon_{k}$ represents the uncertainty in the *k*th grid box, while $w_{k}$ corresponds to the cosine of the latitude. $N_{\text{nom}}^{*}$ represents the nominal number of spatial degrees of freedom, and $N_{\text{eff}}^{*}$ is the corresponding effective number, which is calculated following equation 5 of Bretherton *et al.* (*84*). Before conducting the analysis, the monthly anomalies of SW_lcld for each grid are multiplied by $\sqrt{w_{k}}$ to reduce the influence of grid geometry (*85*). The resulting ${∆}_{\text{susceptibility}}$ represents the half width of the 90% confidence interval for SW ACI, specifically reflecting the uncertainty associated with regression coefficients of susceptibility.

To estimate the uncertainty derived from the aerosol concentration trend of ln(X) , we apply the method described by Santer *et al.* (*18*), which accounts for autocorrelation in the data, ${∆}_{\text{trend}}$ . Thus, the overall 90% confidence interval is expressed as follows

$$\text{SW}\;\text{ACI}\pm \sqrt{{∆}_{\text{susceptibility}}^{2}+{∆}_{\text{trend}}^{2}}$$ (8)
