# Supplementary material for: Negligible contribution from aerosols to recent trends in Earth’s energy imbalance
Source: Sci Adv. 2025 Nov 28;11(48):eadv9429. doi: 10.1126/sciadv.adv9429 (PMC12662203; doi:10.1126/sciadv.adv9429)
Supplement: Supplementary file 1 — Figs. S1 to S12 [file sciadv.adv9429_sm.pdf]

Science Advances
AAAS

Supplementary Materials for

Negligible contribution from aerosols to recent trends in Earth's energy imbalance

Chanyoung Park and Brian J. Soden

Corresponding author: Chanyoung Park, chanyoung.park@miami.edu

Sci. Adv. **11**, eadv9429 (2025)
DOI: 10.1126/sciadv.adv9429

This PDF file includes:

Figs. S1 to S12

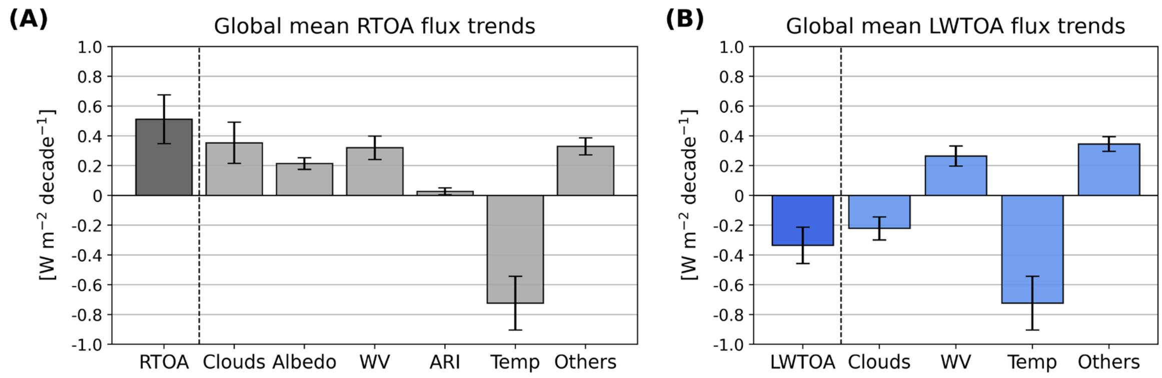

Fig. S1.

Decomposition of global mean top-of-atmosphere (TOA) flux trends from 2003 to 2023, focusing on net TOA radiation (RTOA) and longwave TOA radiation (LWTOA). (A) Global mean RTOA flux trends and their contributions from clouds, surface albedo, water vapor (WV), aerosol-radiation interactions (ARI), temperature, and other factors (labeled as "Others", such as trace gases and solar irradiance). **(B)** Global mean LWTOA flux trends, with contributions from the same components, excluding surface albedo and ARI. Error bars represent the 5–95% confidence intervals determined using the methodology in Santer *et al.* (18). Positive anomalies indicate that the Earth is absorbing more energy, while negative anomalies represent energy loss.

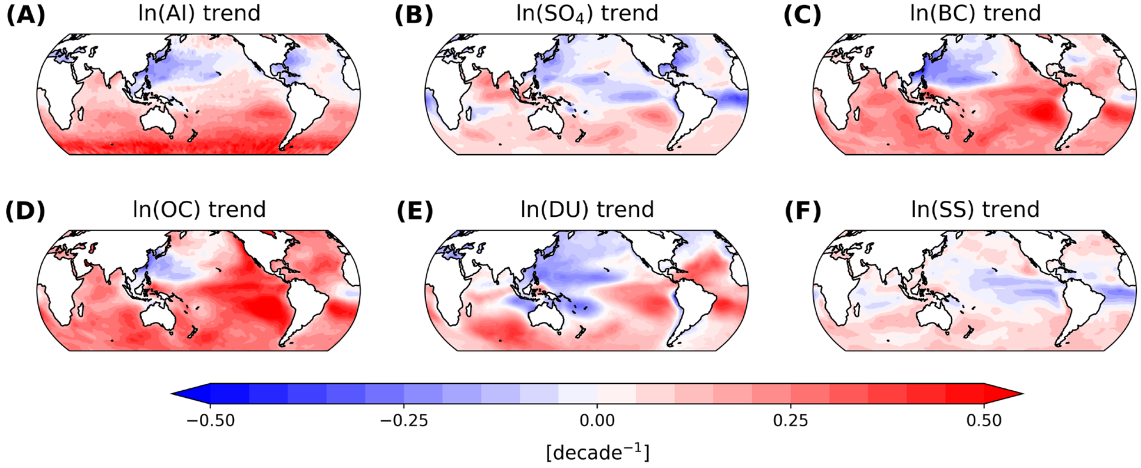

Fig. S2.

Decadal trends (2003–2023) in the natural logarithm of aerosol types. (A) Aerosol index (AI) from MODIS. **(B-F)** Aerosol mass concentrations at 925 hPa from MERRA-2 reanalysis: **(B)** Sulfate (SO_4), **(C)** hydrophilic black carbon (BC), **(D)** hydrophilic organic carbon (OC), **(E)** dust (DU), and **(F)** sea salt (SS). For dust and sea salt, only the smallest size bins (0.1–1 μm for DU and 0.03–0.1 μm for SS) are included to better represent the fine-mode particles most relevant to cloud interactions.

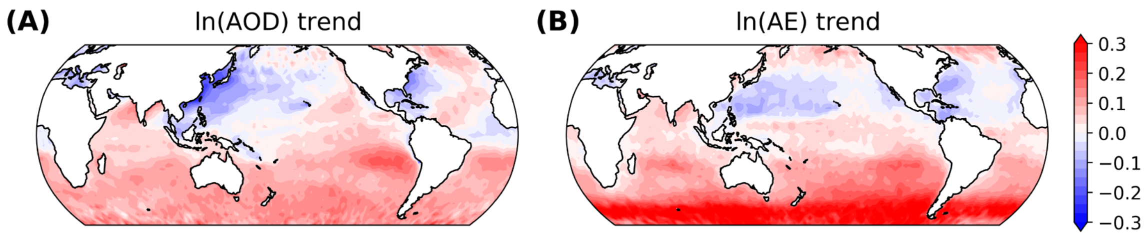

Fig. S3.

Decadal trends (2003–2023) in the natural logarithm of AI components. (A) aerosol optical depth at 550 nm (AOD) and **(B)** the Ångström exponent (AE). AOD captures total aerosol loading, while AE reflects the wavelength dependence of AOD, with a decreasing AE indicating a shift toward larger particles. Together, these components constitute the aerosol index (ln(AI) = ln(AOD) + ln(AE)).

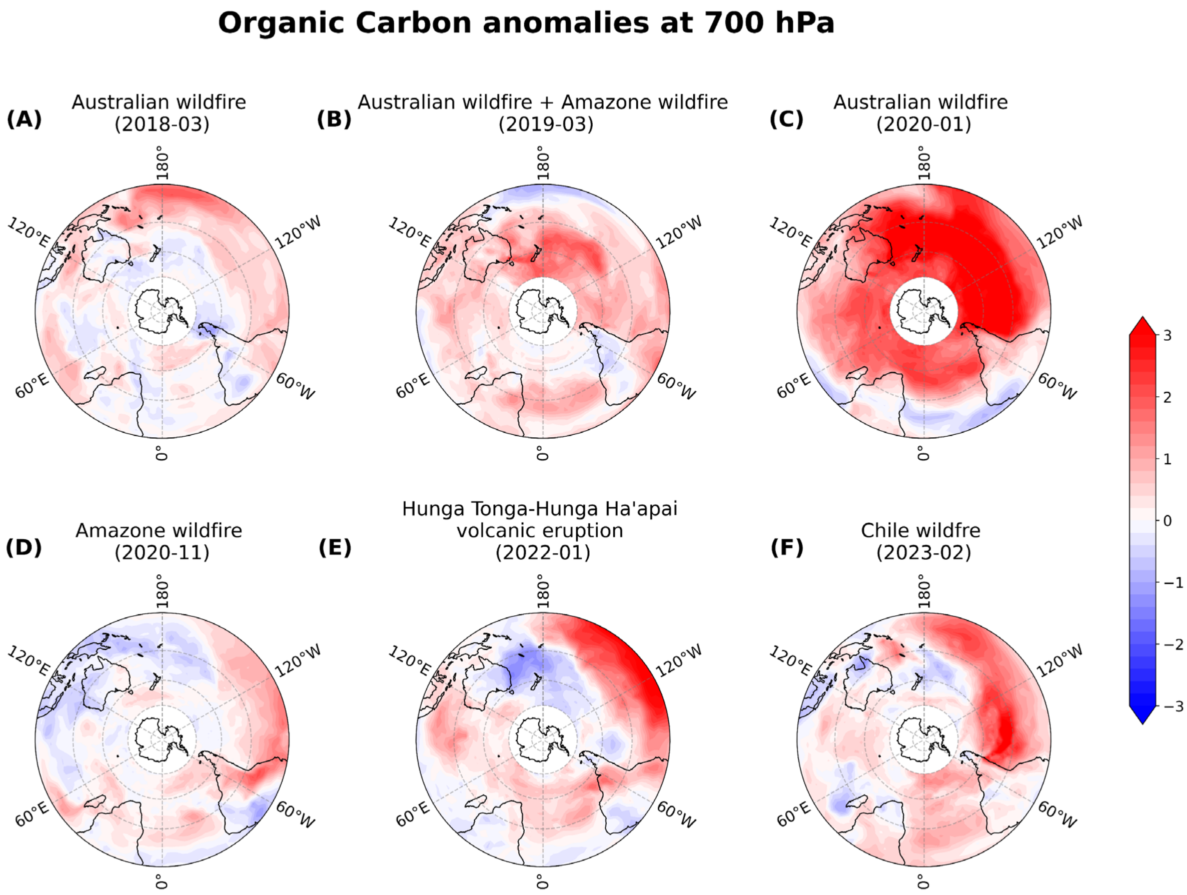

Fig. S4.

Natural logarithm of hydrophilic organic carbon anomalies at 700 hPa during multiple wildfire and volcanic events. Each panel represents a major aerosol emission event in a specific month in the Southern Hemisphere (SH) and the corresponding anomaly: **(A)** Australian wildfire (March 2018), **(B)** combined Australian and Amazon wildfires (March 2019), **(C)** Australian wildfire (January 2020), **(D)** Amazon wildfire (November 2020), **(E)** Hunga Tonga-Hunga Ha'apai volcanic eruption (January 2022), and **(F)** Chile wildfire (February 2023). The anomalies illustrate the impact of these events on atmospheric organic carbon concentrations at 700 hPa. Organic carbon is used in this analysis because it is a primary component of aerosols emitted from biomass burning and can also be influenced indirectly by volcanic activity through interactions with organic precursors, making it a useful tracer for these events.

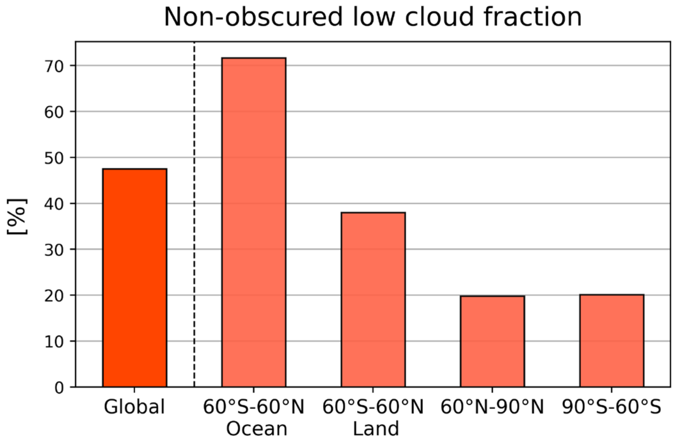

Fig. S5.

Climatological non-obscured low cloud fraction over four regions from 2003 to 2023. Regions include 60°S–60°N over ocean (the primary domain used in this study), 60°S–60°N over land, 60°N–90°N, and 90°S–60°S.

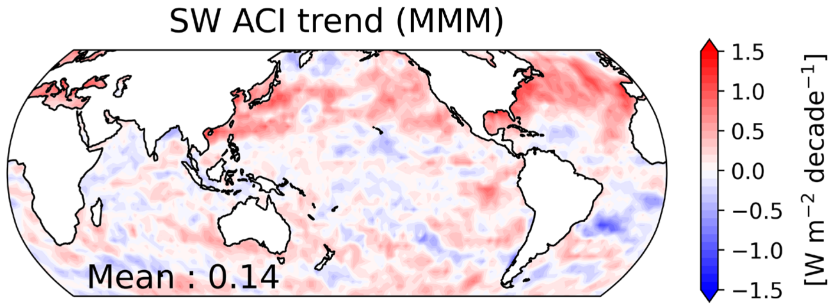

Fig. S6.

Spatial distribution of the decadal trend in shortwave (SW) effective radiative forcing from aerosol-cloud interactions (ACI) for the period 2003–2023. The multi-model mean (MMM) is derived from five models participating in the Radiative Forcing Model Intercomparison Project (RFMIP) (49) single-forcing (aerosol-only) experiments. For this analysis, all models and their realizations, as presented in Fig. 4, are averaged. The domain-averaged (60°S–60°N, ocean) SW ACI trend is displayed in the lower left corner.

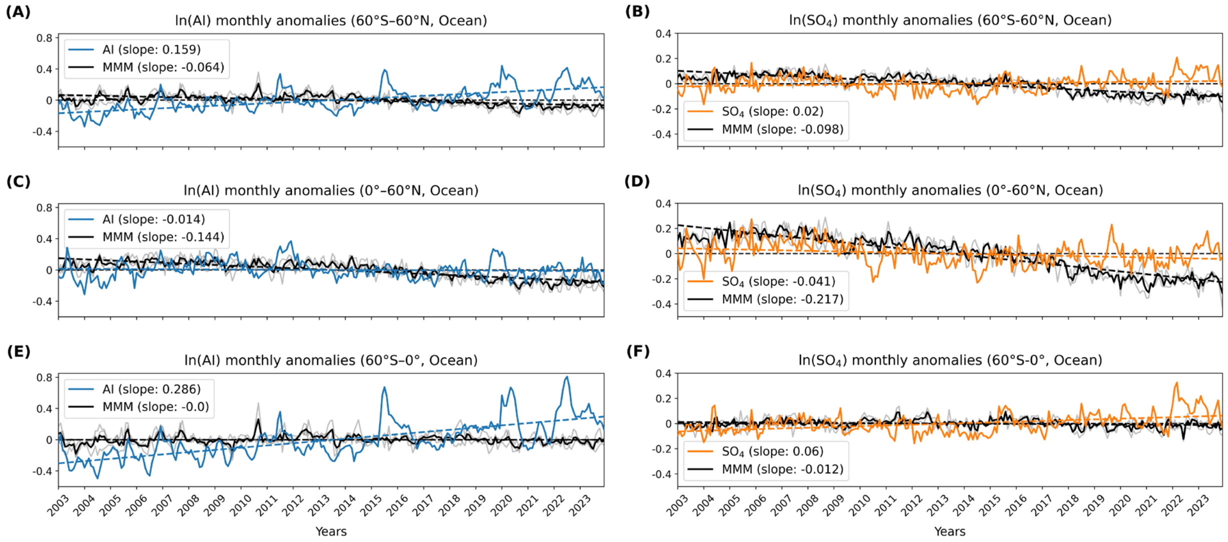

Fig. S7.

Monthly anomalies in the natural logarithm of aerosol index (AI) and sulfate mass concentrations at 925 hPa (SO_4) from observations, reanalysis and CMIP6 models from 2003 to 2023. Anomalies are shown over ocean for three domains: **(A,B)** near-global (60°S–60°N), **(C,D)** Northern Hemisphere (NH; 0°–60°N), and **(E,F)** Southern Hemisphere (SH; 60°S–0°). The left panels display AI anomalies, and the right panels display SO_4 anomalies on a natural logarithmic scale. CMIP6 results include the multi-model mean (MMM, black) and individual model outputs (grey), derived from historical experiments up to 2014, followed by the SSP2-4.5 scenario through 2023. Only models among the five shown in Fig. 4 that provide aerosol proxies, along with their first realization (r1), are included in this analysis: 'HadGEM3-GC31-LL', 'IPSL-CM6A-LR', and 'MIROC6' for AI, and 'GFDL-CM4' and 'MIROC6' for SO_4. Dashed lines represent the linear trends for each component, with their slopes indicated in the upper or lower left corners of each panel.

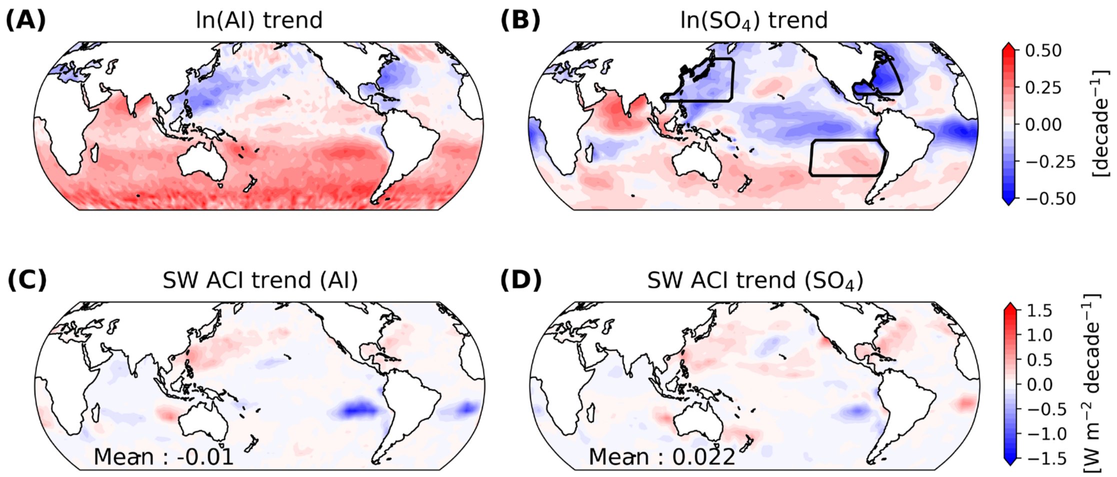

Fig. S8.

Spatial distributions of decadal trends in aerosol proxies, and observationally constrained SW ACI trends from 2003 to 2018. (A) Trends in the natural logarithm of the aerosol index (AI) and **(B)** sulfate aerosol mass concentration at 925 hPa (SO_4). **(C)** Observationally constrained SW ACI trend for AI. **(D)** Same as **(C)**, but for SO_4. The domain-averaged (60°S–60°N, ocean) SW ACI trends are shown in the lower left corners of each panel.

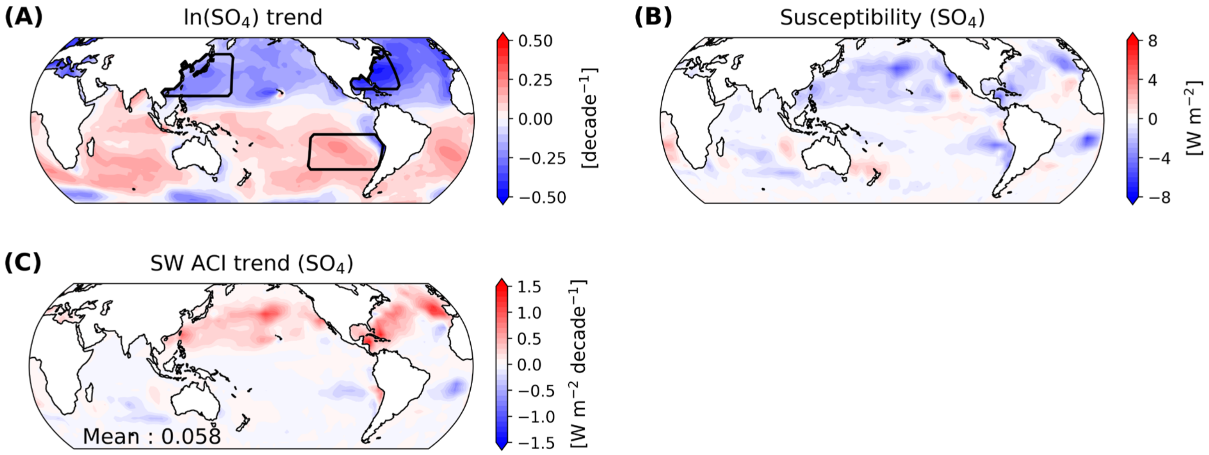

Fig. S9.

Spatial distributions of decadal trends of sulfate mass concentration at 925 hPa (SO_4), susceptibility, and observationally constrained SW ACI trends from 2003 to 2023, using the SO_4 dataset from the CAMS reanalysis. **(A)** Trend in the natural logarithm of SO_4. **(B)** Susceptibility calculated with SO_4 from CAMS reanalysis dataset. **(C)** Observationally constrained SW ACI trend for SO_4. The domain-averaged (60°S–60°N, ocean) SW ACI trend is shown in the lower left corner.

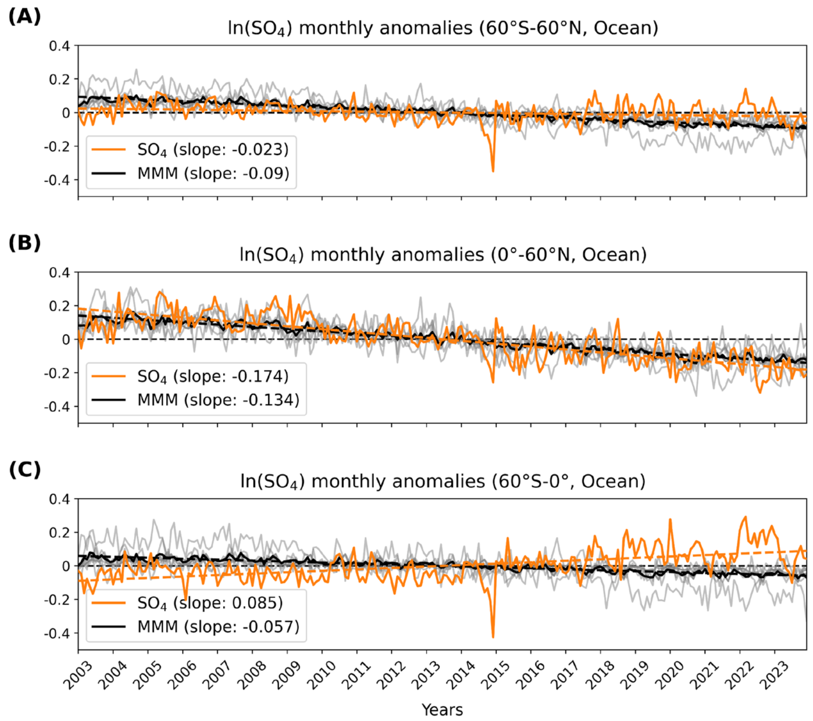

Fig. S10.

Monthly anomalies in the natural logarithm of sulfate mass concentrations at 925 hPa (SO_4) from CAMS reanalysis and CMIP5 models from 2003 to 2023. Anomalies are shown over ocean for three domains: **(A)** near-global (60°S–60°N), **(B)** Northern Hemisphere (NH; 0°–60°N), and **(C)** Southern Hemisphere (SH; 60°S–0°). CMIP5 results include the multi-model mean (MMM, black) and individual model outputs (grey), based on the first realization (r1) of eight models. Historical simulations are used up to 2005, followed by the RCP8.5 scenario through 2023. Models included in the analysis are: 'IPSL-CM5A-LR', 'IPSL-CM5A-MR', 'IPSL-CM5B-LR', 'MIROC5', 'MIROC-ESM-CHEM', 'MIROC-ESM', 'NorESM1-ME ', and 'NorESM1-M'. Dashed lines represent the linear trends for each component, with their slopes indicated in the lower left corners of each panel.

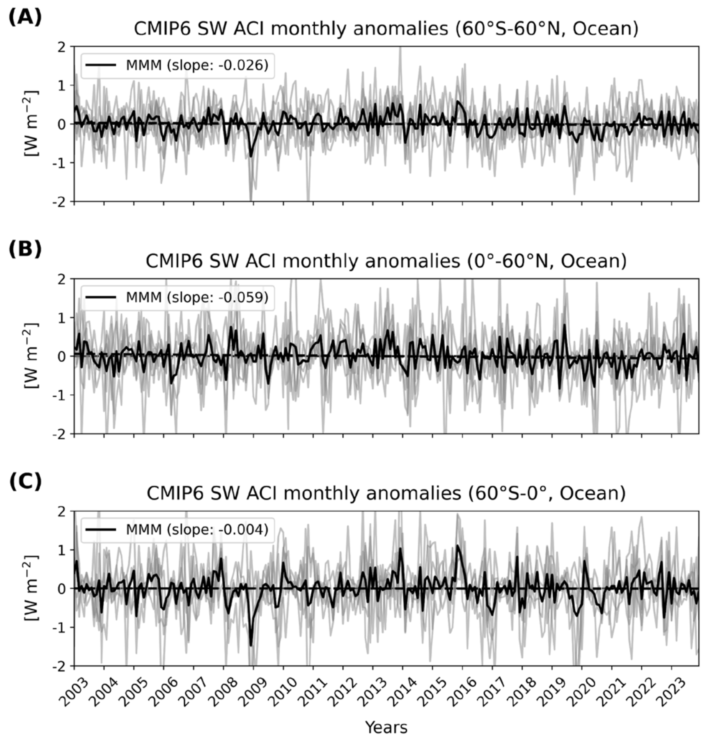

Fig. S11.

Monthly anomalies of SW ACI from CMIP6 RFMIP single-forcing (natural-forcing-only; piClim-histnat) experiments (MMM in black, individual models in grey) over oceans across three different domains from 2003 to 2023. Anomalies are shown over ocean for three domains: **(A)** near-global domain (60°S–60°N), **(B)** Northern Hemisphere (NH; 0°–60°N), and **(C)** Southern Hemisphere (SH; 60°S–0°). The analysis includes five models shown in Fig. 4, each using the first realization (r1). Dashed lines represent the linear trends, with the slopes noted in the upper left corners of each panel.

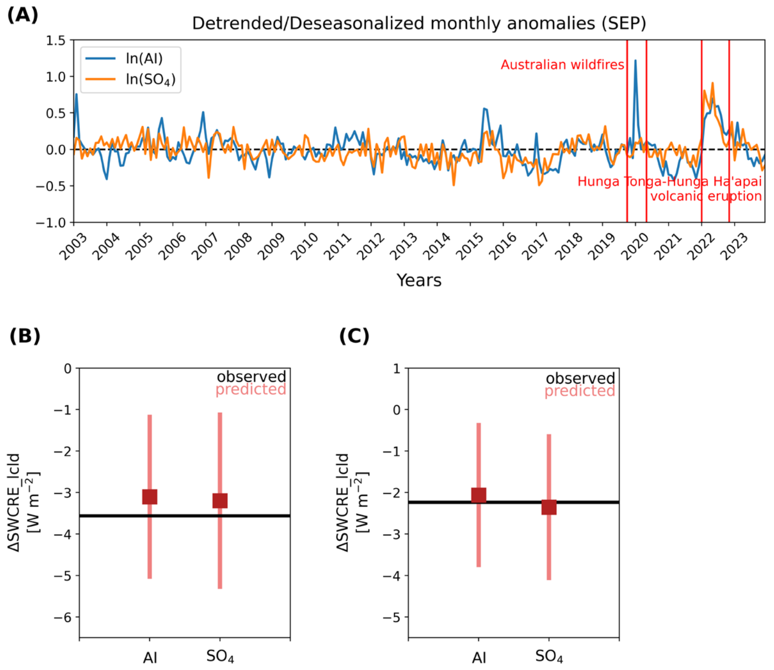

Fig. S12.

Opportunistic experiments validating the methodology of Park *et al.* (38) for two extreme episodic aerosol emission events. (A) Detrended and deseasonalized monthly anomalies of ln(AI) and ln(SO_4) averaged over the southeastern Pacific (SEP; region as shown in Fig. 2). Vertical red lines indicate the timing of the 2019-2020 Australian wildfires (October 2019 to May 2020) and the 2022 Hunga Tonga-Hunga Ha'apai volcanic eruption (January to November 2022). (B) Observed and predicted anomalies in SW_lcld averaged over the wildfire period. (C) Same as (B), but for the volcanic eruption period. Here, ΔSW_lcld refers to the anomalies averaged over each respective episodic event period. Predicted values are the total prediction, including the contribution from changes in aerosol concentration and from changes in environmental factors. Error bars represent 90% confidence intervals.
